# Importance of the Farm Environment and Wildlife for Transmission of *Campylobacter jejuni* in A Pasture-Based Dairy Herd

**DOI:** 10.3390/microorganisms8121877

**Published:** 2020-11-27

**Authors:** Delphine Rapp, Colleen Ross, Shen-Yan Hea, Gale Brightwell

**Affiliations:** 1 Food & Bio-based Products, AgResearch, Hopkirk Research Institute, Tennent Drive, Massey University, Palmerston North 4442, New Zealand; colleen.ross@agresearch.co.nz (C.R.); gale.brightwell@agresearch.co.nz (G.B.); 2 Bioinformatics & Statistics, AgResearch, Grasslands Research Centre, Tennent Drive, Palmerston North 4410, New Zealand; shen.hea@agresearch.co.nz; 3 New Zealand Food Safety Science & Research Centre, Hopkirk Research Institute, Tennent Drive, Massey University, Palmerston North 4442, New Zealand

**Keywords:** *C. jejuni*, dairy, environment, reservoirs, birds, MLST

## Abstract

Cattle are an established reservoir of the foodborne bacterial pathogen *Campylobacter jejuni*. Our six-month study aimed to evaluate sources and pathways governing long-term presence of *C. jejuni* in a pasture-based dairy herd. *C. jejuni* was detected in all sample types (soil, pasture, stock drinking water, bird, rodents and cow faeces). It was persistently detected from cow (54%; 49/90 samples) and bird (36%; 77/211) faeces. Genetic comparison of 252 *C. jejuni* isolates identified 30 Multi-Locus Sequence Types (ST). ST-61 and ST-42 were persistent in the herd and accounted for 43% of the cow isolates. They were also detected on pasture collected from fields both recently and not recently grazed, indicating that grazed pasture is an important pathway and reservoir for horizontal transmission among cows. ST-61 accounted for 9% of the bird isolates and was detected at four of the six sampling events, suggesting that bird populations might contribute to the cycling of ruminant-adapted genotypes on-farm. Overall, the results indicated that management of grazed pasture and supplementary feed contaminated by bird droppings could be targeted to effectively reduce transmission of *C. jejuni* to dairy herds, the farm environment and ultimately to humans.

## 1. Introduction

*Campylobacter jejuni* (*C. jejuni*) is an important bacterial pathogen responsible for acute gastroenteritis worldwide [1]. The burden of *Campylobacter* infections is substantial; campylobacteriosis accounts for about 1.5 million cases of infectious illness in the United States annually [2] and since 2005 has been the most reported gastrointestinal bacterial pathogen in the European Union [3]. In Africa, Asia, and the Middle East, *Campylobacter* infections appear to be endemic, particularly in young children [4]. Post-infection neuropathies can be severe and include Guillan-Barré and Miller Fisher syndromes [4].

*C. jejuni is* widely distributed in most warm-blooded animals, with chickens and cattle identified as the predominant sources from which transmission to humans can occur [5,6]. The transmission pathway to humans is foodborne, via consumption of undercooked poultry, raw milk or vegetables contaminated with animal faeces or effluents [7,8,9]. Direct contact with dairy animals [10] or contact with contaminated recreational waters [11] have also been identified as risk factors for campylobacteriosis. The role of cattle in human infection is further supported by an increased risk of campylobacteriosis in rural areas [12], during flooding of vegetable fields [13] and with periods of high surface-water overland flow combined with cattle grazing [14]. 

One of the strategies to prevent the disease caused by *C. jejuni* is incorporation of control measures at the primary source (i.e., the animal reservoir) [15]. In New Zealand and Iceland, good hygiene and biosecurity measures have proven to be effective in closed housing production systems used for poultry [16]. However, if open pasture is used for grazing cattle, environmental control measures are more challenging. Previous studies have established the prevalence and genotypes of enteric bacteria, such as *C. jejuni*, in dairy cows and their environment, including livestock drinking water [17,18,19], wild birds and mammals living in close proximity to the livestock [20,21]. There is however limited longitudinal data on contamination of multiple farm environments and reservoirs with cattle shedding these pathogens in their faeces, which is required for herd-level epidemiology. 

The objective of the present study was to identify the sources and pathways governing the persistence of *C. jejuni* in a dairy herd by establishing (i) the temporal variation in the occurrence of *C. jejuni* isolated from farm animals, wildlife and environment, and (ii) the genetic relatedness of *C. jejuni* isolates.

## 2. Materials and Methods

### 2.1. Study Site 

The study was performed on a commercial dairy farm in a temperate region (Waikato district) of New Zealand. A herd of 454 cows was farmed on 83 ha of farmland. The cows were grazed on pasture throughout the study and received supplementary feed (including palm kernel, maize silage, and soya meal). Feed was kept in open barns or bunkers and was partially covered. When not on pasture, the cows were kept on two 620 m^2^ wall-free shelters comprising a clear roof, slatted concrete floors and an under-floor manure bunker. These facilities were used for 2–10 h per day by the cows. Stock drinking water was sourced from a bore. 

### 2.2. Sampling Collection

Samples were collected from the farm once a month for six months (May to October, covering winter and spring seasons). At each visit, cow fecal samples (50–200 g) were collected from faeces freshly voided on a field that was grazed for up to 12 h before sampling. Cross-contamination during sampling was avoided using single-use plastic spoons. Environmental samples were collected from the field the cows were currently in and from the field to be grazed next (no grazing or irrigation with farm dairy effluents for 21 days). For each field, a composite pasture sample (about 300 g) was collected by bulking herbage from 15 sites at evenly spaced intervals along one diagonal transect line, avoiding dung and urine patches. Herbage was clipped at approx. 5 cm above ground level using sterile scissors and placed in a clean plastic bag. A composite soil sample (approx. 150 g) was collected by bulking a total of 15 soil cores (diameter 2  cm and soil depth 0–5  cm) manually excavated at the pasture collection sites by using a stainless-steel soil corer. For each field, a one-litre water sample was collected by immersing a sample bottle in the top layer of the water column of the livestock drinking water. At each visit, a one-litre livestock drinking water sample was also collected from the animal shelters using the same procedure. At each visit, up to 45 individual moist bird droppings were collected from the fences, rails and edges of the concrete feed bunkers and cow shelters. Single droppings were transferred to a screw lid vial using sterile tweezers. When found in the area of supplementary feed and animal shelter, rat faeces were collected using sterile tweezers and placed as single pellets into screw-lid vials. All samples were placed in an insulated box, transported to the laboratory and analysed within 6 h of collection.

### 2.3. Cultivation and Identification of C. jejuni

The presence of *C. jejuni* was determined from a representative subsample of each solid sample using selective enrichment in *Campylobacter* modified Exeter broth (mExBr) followed by a secondary selection on mCCDA agar as described in the NZ Reference method [22]. Ratios in mExBr were 1:30 (*w/v*) for cow faeces, 1:10 (*w/v*) for soil samples, 1:5 (*w/v*) for pasture. Five hundred millilitres of each trough water sample was filtered through 0.45 µm filters, which were then placed in 75 mL mExBr. More than one filter was required if samples were turbid. Each individual bird dropping or rat faeces was added directly to 10 mL of mExBr. After 48 h incubation at 42 °C, a 20 μL volume of the enriched mExBr was transferred to a mCCDA plate and grown in a microaerophilic atmosphere at 42 °C for 24 h. The presence of *C. jejuni* on mCCDA was confirmed by PCR with a primer pair specific for *C. jejuni* [23]. Bacterial cells were stored in charcoal Amies transport medium (Fort Richard Laboratory Ltd., Auckland, New Zealand) at −80 °C until purification of single-colony isolates for genotyping. 

### 2.4. Genotyping of C. jejuni Single-Colony Isolates by Enterobacterial Repetitive Intergenic Consensus (ERIC)-PCR and Multi Locus Sequence Typing (MLST)

Up to two single colonies were genotyped for each *C. jejuni*-positive sample. Resuscitation of the cells stored in charcoal Amies transport medium and isolation of single colonies were performed using the conditions described above. DNA purification and ERIC-PCR analyses were performed as previously described [24]. The ERIC profiles were compared in two stages. For each of the six sampling visits, the DNA of single-colony isolates from each individual sample was first compared to each other to determine the occurrence of ERIC types at a sampling time. DNA of representative single-colony isolates from each sampling was then successively compared with those of the other sampling visits. Profiles were assigned a numerical sequence applied randomly (i.e., ERIC-type 1 to ERIC-type 30). *C. jejuni* isolates (*n* = 37) representative of the ERIC types obtained during the study were further characterized by MLST for their association with human infections and with other animal reservoirs. MLST analyses were conducted by mEpiLab (Hopkirk Research Institute, Massey University, Palmerston North, New Zealand). Sequence data were collated, and alleles assigned as sequence type (ST) and clonal complex (CC) using the *Campylobacter* PubMLST database (http://pubmlst.org/campylobacter/).

### 2.5. Statistical Analysis

A generalized linear model with a logit link function was used to investigate the relationship between the proportion of *C. jejuni* positive samples and the explanatory variables sampling month and sample type. Both explanatory variables were included in the model as factors. Rarefaction curves were created using the Vegan R package [25,26] to assess *C. jejuni* population diversity in the cows and the birds. The R software program was used for data manipulation and statistical analysis.

## 3. Results

### 3.1. Prevalence of C. jejuni in the Cows and the Farm Environment

The overall prevalence of *C. jejuni* in the samples collected over a 6-month period at the study farm was 42% (154/369 samples) (Table 1). *C. jejuni* prevalence varied among sample types as well as between sampling months (*p* < 0.001). *C. jejuni* was detected in both cow faeces and bird droppings at each sampling occasion, at prevalences of 54% (49/90 samples) in cows and 36% (77/211) in birds. The highest rates of prevalence were in Spring, in both August and September for cows and in September for birds. *C. jejuni* was also found in rat faeces (6/7 samples), which were found only at the August and September samplings. In fields recently (<24 h) grazed by the cows, *C. jejuni* was repeatedly detected in pasture (69%, 9/13 samples), soil (38%, 5/13) and livestock drinking water (21%, 4/19). In the fields not recently (>21 days) grazed or effluent-irrigated, it was found in the pasture samples at four of the six samplings occasions but was not detected in the soil (0/6) or livestock drinking water (0/5) samples. 

### 3.2. Characterization of C. jejuni Isolates

A total of 252 *C. jejuni* isolates were obtained from the animal and environmental samples. All the isolates were characterised by ERIC-PCR into 30 distinct ERIC types at a similarity cut-off value of 90%. The most prevalent ERIC types were types 1 (*n* = 40, 16%), 2 (*n* = 34, 13%) and 6 (*n* = 35, 14%), followed by types 17 (*n* = 22, 9%), 12 (*n* = 19, 7%) and 13 (*n* = 16, 6%). The remaining 24 ERIC-types collectively represented 35% of the entire data set. MLST analysis identified 12 *C. jejuni* sequence types (STs) grouped into 10 clonal complexes (CCs). One MLST sequence type from bird isolates has not been previously assigned (ERIC type 17). Four representative ERIC types (23, 8, 2 and 18) were unidentified by MLST.

#### 3.2.1. Ruminant Isolates

A total of 14 ERIC-types representative of seven sequence types were identified from the cow isolates dataset (*n* = 87) (Table 2). Two ERIC-types (1 and 6) were the most common in the cows, accounting for 43% of the total cow isolates and detected on five of the six sampling occasions (Table 3). These two types belonged to MLST ST-61 and ST-42, respectively. ERIC types (2 and 17), which were assigned to new STs, were also common in the cows (15 and 10% of cow isolates; detected on 2 and 5 occasions). ERIC type 13, assigned to ST-38, represented 10% of the cow isolates. The remaining 9 ERIC types, representing six sequences types, occurred only once in the cow dataset.

#### 3.2.2. Wildlife Isolates

**Figure 1:**
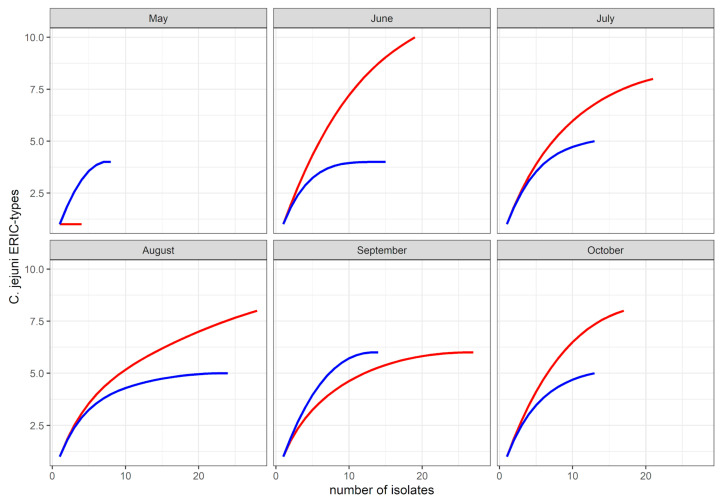
Rarefaction curves of *C. jejuni* population in cow faeces (blue) and wild bird droppings (red) during the study period.

From the bird dataset (*n* = 116 isolates), a total of 25 ERIC-types, representative of 13 sequence types, were identified. Comparison of *C. jejuni* genetic diversity by sample type-based rarefaction curves revealed a greater ERIC-type diversity in bird isolates compared to bovine isolates (Figure 1). ERIC-types 2 and 12 (ST-45) were the most commonly detected in the birds. They were represented by 17% and 13% of the bird dataset, and were found at three and four sampling occasions, respectively. The ERIC-types 6 (ST-42), 17 (ST-10821) and 27 (ST-45) were represented by 10, 9 and 7 isolates, respectively (9 to 6% of the bird dataset). Both ERIC-types 6 and 27 were stable in the bird population, each detected on four of the six sampling times. The remaining 20 ERIC-types were represented by six isolates or less (<5% of the bird dataset). They were often detected once in the birds during the study. The exceptions were ERIC-type 10 (ST-45), which was detected at three sampling occasions, and ERIC-types 1 (ST-61), 9 (ST-2026) and 13 (ST-38), which were detected on two sampling occasions. Of the 20 ERIC-types detected from time to time in the bird dataset, six were shared with the cow dataset. Of the seven ERIC types that were assigned to sequence type ST-45, five were detected in the bird dataset, in which they accounted for a total of 29% of the bird dataset. From the rat faeces (*n* = 9 isolates), two ERIC-types (12 and 30) were identified, both assigned to ST-45.

#### 3.2.3. Farm Environment Isolates

The environmental isolates (*n* = 40) were obtained from fields recently grazed or effluent irrigated, as well as from fields free from fresh faecal deposits from dairy cows. In total, nine ERIC-types were identified, representative of 8 sequence types, of which one was new, and one was unassigned. The most commonly encountered ERIC-type was ERIC-type 31 (17 isolates; 32% of the environmental dataset), which was representative of ST-50. This type was detected at a single sampling visit (October); it was not detected in the cows during the study. Two other types commonly detected in the farm environment were ERIC-type 1 (14 isolates, 26% of the environmental dataset) and ERIC-type 6 (8 isolates, 15% of the environmental dataset). Four ERIC-types (1, 2, 3 and 6) were detected in fields not recently grazed by the cows or irrigated with animal effluents; they were each detected at one or two occasions in pasture.

## 4. Discussion

Establishing the occurrence and genetic diversity of zoonotic organisms in livestock farms can provide information on transmission dynamics, persistence or emergence of zoonosis. The results of our longitudinal dataset confirmed the temporal persistence and high prevalence of *C. jejuni* in dairy farms, as previously reported [24]. Analysis of the genotypic composition of the isolated *C. jejuni* population by comparative multilocus sequence typing (MLST) revealed that the most abundant and stable genotypes in the studied herd were ST-61 and ST-42 complexes. The observed stability of some genotypes in adult cattle herds over time is consistent with other longitudinal studies [20,27,28]. It has been attributed to a small number of *C. jejuni* sources, adaptation of particular MLST types and transmission of the organism among animals that are held in close proximity [17,29,30]. Worldwide, the ST-61 and ST-42 complexes have been identified as examples of specialist lineages associated with cattle [31]. They also represent an important component of the genotypes known to be associated with ruminants in New Zealand [6,32], supporting the hypothesis that these ruminant-adapted genotypes are readily transmitted in a dairy herd. Taken together, our findings suggest that cow to cow transmission is important for persistence of ruminant-adapted *C. jejuni*.

Several routes have been proposed for dissemination of *C. jejuni* on dairy farms, including direct animal contact and mutual grooming [33], and aerosolization or splatter of faeces onto surfaces such as boots, tractors or even silage [20,34]. As expected, the *C. jejuni* genotypes detected in the environment, principally in recently grazed fields, were similar to those detected in the cows, confirming that pasture, soil and water contaminated with cow faeces may contribute to indirect horizontal transmission [17,35]. The chosen sampling protocol, which was designed to analyse a representative sample for each sample type, revealed a high prevalence of *C. jejuni* on pasture, implying a high risk of exposure and ingestion of *C. jejuni*. The composite sampling design may however have influenced our ability to detect *C. jejuni* in the soil underneath sampled pasture and in drinking trough water. It is possible that contamination of soil was heterogeneous at the scale of a field, as it has been seen with other zoonotic bacteria [36] and that differences in prevalence among the different sample types could be due to the sample size or physical properties affecting the success of the homogenization step. Pasture has not been previously highlighted as an important vector for transmission of *C. jejuni* among ruminants, but it has been reported that dairy cows would prefer to consume vegetation not contaminated with fresh faeces [37]. The analysis of a larger number of environmental samples from a field, as well as determination of pasture consumption behaviours are recommended to identify environmental hot spots and to more accurately quantify the role of pasture, soil, and water to maintain *C. jejuni* prevalence in dairy herds.

It has been proposed that a change of fields can shift a zoonotic population over time in ruminant herds due to the introduction of new genotypes [38]. In our study where different fields were analysed, the ruminant-associated genotypes ST-61 and ST-42 were occasionally isolated from pastures that had not been recently grazed or irrigated with animal effluents. The presence of ST-61 and ST-42 in these fields is in contradiction with a study in a 100-km^2^ farmland in England, which reported that ruminant genotypes were the least likely to be found in the environment [27]. Different farming systems, paddock rotation practices or seasonal prevalence might explain the discrepancies between the two studies, but our data suggests that the number of ruminant-associated *C. jejuni* cells that were initially shed on pasture is important for environmental persistence and re-infection of the dairy herd during the next grazing period. This hypothesis is supported by the common presence of ST-61 and ST-42 in cow faeces where *C. jejuni* concentration was greater than 3 log_10_ per g (fresh weight) [24]. Faecal loadings and environmental concentration of specific genotypes are warranted in further epidemiological studies.

Frequent contact opportunities between wildlife and farmed animals can facilitate pathogen spill over from wildlife to livestock and vice versa [39], and many epidemiology studies have discussed *C. jejuni* transmission between wild birds and livestock [20,34,40]. While the available literature has focused on bird species in a large range of habitat settings such as urban areas [41], feedlots [42], and large geographical areas [43], our longitudinal monitoring provided insight into the dynamics of *C. jejuni* populations between ruminant and birds on a farm. The bird community was *C. jejuni*-positive for the entire duration of the study, with an overall prevalence similar to that previously reported [20,44]. The bird population also carried a wide range of *C. jejuni* strains, as observed in other farm studies [44,45]. The prevalence and diversity of *C. jejuni* in the wild bird population has been associated with the bird taxa and feeding habits [46]. In our study, large flocks of house sparrows *(Passer domesticus)*, silvereyes (*Zosterops lateralis)*, Indian mynas (*Acridotheres tristis*) and starlings (*Sturnus vulgaris*) were observed foraging in the supplementary feed and water troughs, as well as around stores of supplementary feed that were left uncovered. A rapid turnover and re-colonization of starlings with different *C. jejuni* genotypes has been reported [45], however the persistent presence of ST-42 and ST-61 (ERIC types 1 and 6) in bird droppings supports a possible role of a “cycling” of “ruminant-adapted” *C. jejuni* between cows and birds for maintaining *C. jejuni* in the herd.

The clonal complex ST-45, classified as a lineage with a generalist lifestyle, has been regularly isolated from multiple species of hosts [31,47]. In the present study, ST-45 (ERIC type 12 and 27) appeared to be abundant and stable in the studied bird population but was infrequently detected in cows, suggesting the possibility of barriers to transmission and establishment of “generalist” *C. jejuni* in this dairy herd. This hypothesis is supported by a similar finding observed for other generalist types the cows were exposed to, for example clonal complexes ST-48, ST-206 and ST-21, and by a longitudinal monitoring of dairy cows, in which ST-45 was detected for a long period of time in a small proportion of “high-shedder” cows, but sporadically for the other studied cows [48]. ERIC genotyping revealed a high genetic diversity within our ST-45 strains. Genomic differences between and within generalist lineages have been associated with phenotypic differences and flexibility in terms of metabolic properties, energy harvests, or cell invasiveness [49,50], and it is possible that only some sub-lineages of ST-45 are able to exclude those genotypes that are particularly recognized to be adapted to the bovine intestine.

## 5. Conclusions

Our study has demonstrated transmission of *C. jejuni* between and within a range of animal species associated with pasture-based dairy farms. It has highlighted the role of environmental pathways for *C. jejuni* contamination of dairy cows. It is clear that grazed pasture can be an important pathway and reservoir for horizontal transmission, and that wild birds foraging on supplementary feed can increase the cycling of ruminant adapted *C. jejuni* genotypes. Through the bird population, cows can be exposed to generalist *C. jejuni,* but there appear to be a complex ecology associated with transmission and establishment of these types within the herd. Further work is needed to design management strategies that might effectively reduce carriage and transmission of *C. jejuni* to dairy herds, the farm environment and ultimately to humans.

## Figures and Tables

**Table 1:** Occurrence of *C. jejuni* in cows, wildlife and farm environment during the study period.

Sample Type	Number *C. jejuni*-Positive Sample/Total Samples (%)						
	May	June	July	Aug	Sept	Oct	Total
Cows faeces	4/15 (27)	8/15 (53)	8/15 (53)	12/15 (80)	10/15 (67)	7/15 (47)	49/90 (54)
Pasture	1/2 (50)	1/3 (33)	2/3 (67)	3/4 (75)	4/4 (100)	2/3 (67)	13/19 (68)
Soil	0/2 (0)	1/3 (33)	1/3 (33)	2/4 (50)	1/4 (25)	0/3 (0)	5/19 (26)
Water	0/3 (0)	0/3 (0)	1/4 (25)	0/5 (0)	1/5 (20)	2/3 (67)	4/23 (17)
Bird droppings	2/12 (17)	13/35 (37)	12/41 (29)	15/45 (33)	25/42 (60)	10/36 (28)	77/211 (36)
Rat faeces	0/0 (0)	0/0 (0)	0/0 (0)	0/1 (0)	6/6 (100)	0/0 (0)	6/7 (86)
Total							154/369 (42)

**Table 2:** Relative distribution of *C. jejuni* ERIC genotypes within cows, birds, and environmental datasets. The rodent dataset (9 isolates) was not included in the table.

ERIC Genotype	Total Number of Isolates	% Isolates within Datasets				MLST
		Cow (*n* = 87)	Birds (*n* = 116)	Environment (Pasture, Soil, Water) (*n* = 40)	Sequence Type	Clonal Complex
1	40	23	5	35	ST-61	CC-61
5	3	2	1	0	ST-61	
6	35	20	9	20	ST-42	CC-42
7	4	4	0	0	ST-42	
29	3	3	0	0	ST-42	
4	1	0	1	0	ST-42	
12	17	2	13	0	ST-45	CC-45
30	4	2	2	0	ST-45	
10	6	0	5	0	ST-45	
27	7	0	6	0	ST-45	
25	3	0	3	0	ST-45	
11	1	1	0	0	ST-45	
20	1	0	0	3	ST-45	
24	1	0	1	0	ST-583	
13	16	10	4	5	ST-38	CC-38
21	5	0	1	10	ST-50	CC-21
3	5	2	2	3	ST-50	
14	5	1	3	0	ST-4337	
22	7	0	3	10	ST-2345	CC-206
19	2	2	0	0	ST-2345	
9	4	0	3	0	ST-2026	CC-403
16	3	0	3	0	ST-2343	CC-48
26	2	0	2	0	ST-508	CC-508
28	2	0	2	0	ST-508	
15	1	0	1	0	ST-682	CC-682
17	22	15	8	0	ST-10821	
23	6	0	5	0	Not identified	
8	1	0	1	0	Not identified	
2	34	10	17	13	Not identified	
18	2	0	1	3	Not identified	

**Table 3:** Monthly detection of *C. jejuni* ERIC genotypes in cows, wildlife and the farm environment during the study period. Each genotype was assigned a numerical sequence (i.e., ERIC-type 1 to ERIC-type 30).

Sample Type	*C. jejuni* ERIC Genotypes					
	May	June	July	Aug	Sept	Oct
Cows faeces	1, 6, 12, 17	1, 6, 13, 17	7, 11, 13, 17, 29	1, 2, 3, 5, 6	1, 2, 6, 13, 17, 30	1, 6, 14, 17, 19
Pasture	1, 13	22	2, 6, 20	2, 3, 6,	1, 2, 13	1
Soil		22	6	1, 6		
Water			1		18	21
Birds droppings	10	4, 6, 8, 9, 12, 18, 22, 25, 26, 27	1, 2, 5, 6, 12, 14, 17, 23	2, 3, 6, 12, 14, 17, 24, 27	1, 2, 6, 13, 27, 30	9, 10, 12, 15, 16, 21, 27, 28
Rat faeces					12, 30	
